# The reliability and diagnostic accuracy of the GAD-7 and GAD-2 for the most prevalent anxiety disorders in Latvian primary care

**DOI:** 10.3389/fpsyt.2026.1855491

**Published:** 2026-07-07

**Authors:** Lubova Renemane, Anda Ķīvīte-Urtāne, Elmārs Rancāns

**Affiliations:** 1 Department of Psychiatry and Narcology, Riga Stradiņš University, Riga, Latvia; 2 Institute of Public Health, Riga Stradiņš University, Riga, Latvia

**Keywords:** anxiety disorders, diagnostic accuracy, GAD-2, GAD-7, primary care

## Abstract

**Introduction:**

Anxiety disorders are common but often under-recognized in primary care. Validation data for the GAD-7 and GAD-2 in Eastern European primary care settings remain limited. To determine the reliability and diagnostic performance of the GAD-7 and GAD-2 for the most prevalent anxiety disorders in Latvian primary care using the Mini International Neuropsychiatric Interview (MINI) as the reference standard.

**Methods:**

Consecutive adult patients from 24 primary care practices completed the GAD-7 and GAD-2 prior to consultation. Within two weeks, blinded psychiatrists conducted MINI interviews by telephone. Internal consistency was assessed using Cronbach’s alpha. Diagnostic accuracy was evaluated using receiver operating characteristic analyses, sensitivity, specificity, and likelihood ratios.

**Results:**

The final analytical sample comprised 1,467 participants. The most prevalent MINI-defined anxiety disorders were agoraphobia (8.0%), generalized anxiety disorder (6.1%), and social phobia (4.7%). Internal consistency was 0.86 for the GAD-7 and 0.72 for the GAD-2. For generalized anxiety disorder, optimal cut-offs were ≥6 (GAD-7; sensitivity 71.9%, specificity 76.3%, AUC = 0.799) and ≥2 (GAD-2; sensitivity 80.9%, specificity 65.8%, AUC = 0.772). GAD-7 and GAD-2 performance for social phobia, agoraphobia and any anxiety disorders was relatively weak. For any anxiety disorder, the optimal thresholds were identified as ≥4 (GAD-7; sensitivity 70.6%, specificity 61.6%, AUC = 0.718) and ≥2 (GAD-2; sensitivity 64.9%, specificity 68.1%, AUC = 0.691).

**Conclusion:**

The GAD-7 and GAD-2 demonstrate satisfactory reliability and the strongest diagnostic accuracy to screen for generalized anxiety disorder in Latvian primary care, whereas performance for other anxiety disorder subtypes was comparatively more limited. Subtypes of anxiety disorders should be considered and specific cut-offs calculated to improve the detection of different anxiety disorders in routine general practice.

## 1 Introduction

For Over the past three decades, the global prevalence of anxiety disorders has increased substantially, with the number of affected individuals rising by more than 55%, making anxiety disorders among the most common mental health conditions worldwide (1). Lifetime prevalence estimates in the general population range from 13.6% to 28.8%, while 12-month prevalence rates are approximately 14% across European and other international settings (2–5). These disorders disproportionately affect women, younger individuals, and socially disadvantaged groups, and they frequently co-occur with depression and substance use disorders (5). In primary care, the burden is particularly pronounced, with point prevalence estimates of anxiety disorders ranging from 25% to 30% (4, 6). Despite this high prevalence, recognition remains suboptimal, as general practitioners identify only about two-thirds of affected patients, and many individuals do not receive minimally adequate treatment (7). The substantial functional impairment and increased healthcare utilization associated with anxiety disorders, underscore the need for reliable and efficient screening strategies in primary care (8).

Several screening instruments have been developed to support the early identification of anxiety disorders. Among them, the seven-item Generalized Anxiety Disorder scale (GAD-7), is the most extensively validated and commonly used instrument for detecting anxiety disorders in both clinical practice and research contexts (9, 10). The GAD-7 is a brief self-report measure that has demonstrated strong reliability and validity across diverse populations, including pregnant women, individuals in substance use treatment, patient with migraine, and adolescents, with internal consistency coefficients typically ranging from 0.77 to 0.91 and acceptable sensitivity and specificity across commonly used thresholds (10–13). The ultra-brief GAD-2, comprising the first two items of the GAD-7, provides high sensitivity and is well suited for rapid case-finding, although its specificity is comparatively lower (13–15). While additional screening tools exist, the GAD-7 and GAD-2 remain practical, scalable, and particularly appropriate for primary care, underscoring the importance of evaluating their diagnostic accuracy within specific cultural and clinical contexts (10).

Despite their widespread international use, evidence on the psychometric properties and diagnostic performance of the GAD-7 and GAD-2 in Eastern European primary care settings remains limited (16). In Latvia, locally validated data on the diagnostic accuracy of these instruments across anxiety disorder subtypes are lacking, despite primary care being the main point of contact for individuals presenting with emotional distress. Given the substantial burden and frequent under-recognition of anxiety disorders in primary care, locally validated screening tools are essential to enhance accurate identification and inform clinical management (10).

This study aims to evaluate the reliability and diagnostic performance of the GAD-7 and GAD-2 for the most prevalent anxiety disorders in a large Latvian primary care sample, using the MINI as the reference standard, thereby strengthening early detection and improving mental healthcare pathways.

## 2 Materials and methods

### 2.1 Procedure and participants

For This study was carried out as part of the National Research Program BIOMEDICINE 2014–2017, which focused on assessing the prevalence of mental disorders in Latvian primary care. The program, funded by the Latvian Ministry of Education and Science, aimed to develop improved approaches for the prevention, diagnosis, and treatment of mental disorders, as well as to enhance biomedical technologies to promote public health in Latvia.

Participants were recruited from 24 primary care practices (16 urban and 8 rural) representing all geographic regions of the country. During a designated one-week recruitment period, all patients aged 18 years or older who consulted their general practitioner (GP) for any health-related concern were invited to participate. Individuals attending for administrative purposes were not eligible. Additional exclusion criteria included refusal to participate, age under 18 years, and inability to take part due to acute medical conditions requiring urgent hospitalization or other significant health limitations (e.g., one patient with deaf-mutism).

All consecutive eligible patients were asked to complete a paper-and-pencil version of the GAD-7 prior to their GP visit. Participants also completed a structured socio-demographic questionnaire.

The MINI, Version 6.0.0, was administered by telephone by four trained psychiatrists who were blinded to participants’ GAD-7 scores (17). The diagnostic interview was conducted within two weeks of the patient’s initial contact with the primary care practice. The MINI served as the reference standard for establishing the presence of GAD and other anxiety disorders.

The study protocol was approved by the Ethics Committee of Riga Stradins University (Approval No. 8/18.06.2015). Written informed consent was obtained from all participants. All study procedures were performed in accordance with the Declaration of Helsinki and its later amendments.

### 2.2 Measures

The GAD-7 is a 7-item self-report instrument designed to assess core symptoms of generalized anxiety and facilitate rapid screening in clinical and research settings. Each item is rated on a 4-point Likert scale ranging from 0 (“not at all”) to 3 (“nearly every day”), reflecting symptom frequency over the preceding two weeks. Total scores range from 0 to 21, with higher scores indicating greater symptom severity. In the original validation study, a cut-off score of 9 yielded a sensitivity of 89% and a specificity of 82% (9).

Two independent professional bilingual translators performed separate forward translations of the GAD-7 into Latvian, and a third independent translator, blinded to the original questionnaire, subsequently conducted the back-translation into English. The translated versions were compared, discussed, and reconciled into a unified Latvian version by a multidisciplinary panel of Latvian-speaking psychiatrists with clinical experience in anxiety disorders. Translation quality was evaluated through expert assessment of semantic, conceptual, and clinical equivalence, as well as comprehensibility and cultural appropriateness of the items and response options. Potential linguistic ambiguities and culturally specific nuances were further assessed in a focus group, and minor wording refinements were introduced where necessary. This translation approach was chosen to preserve the conceptual meaning of the original instrument and maximize cross-cultural applicability prior to psychometric validation. The GAD-2 comprises the first two items of the GAD-7 and provides an ultra-brief measure for detecting anxiety symptoms. Scores range from 0 to 6. Initial validation work demonstrated a sensitivity of 86% and a specificity of 83% at a cut-off score of 2 (6).

The MINI is a structured diagnostic interview based on criteria from the Diagnostic and Statistical Manual of Mental Disorders and the International Classification of Diseases, 10th Revision (17). It is widely used in psychiatric epidemiology and primary care research and has been translated and adapted into 67 languages, including Latvian (18, 19). The interview contains 120 items and screens for 17 Axis I disorders, yielding 24 current and lifetime diagnostic categories.

In this study, the MINI was conducted by telephone, a method validated in previous research, and all diagnostic modules were administered (20). Current diagnoses of anxiety disorders were identified, including panic disorder, agoraphobia, social phobia, obsessive-compulsive disorder (OCD), posttraumatic stress disorder (PTSD), and generalized anxiety disorder (GAD). For analytic purposes, “any anxiety disorder” was defined as the presence of at least one current MINI-defined anxiety diagnosis.

Participants self-reported sex, age (years), marital status, employment status, educational attainment, and place of residence. For analysis, age was categorized (18–34, 35–49, 50–64, ≥65 years). Marital status was grouped as married/cohabiting, single, or separated/divorced/widowed; employment status as employed, unemployed, or economically inactive; educational attainment as higher/unfinished higher, secondary (general/vocational, including unfinished), or basic/unfinished basic; and residence as urban (Riga or other city) or rural.

Internal consistency of the GAD-7 and GAD-2 was evaluated using Cronbach’s alpha coefficients. Item-level psychometric properties of the Latvian GAD-7 were additionally examined using corrected item–total correlations, Cronbach’s alpha if item deleted, scale mean if item deleted, and scale variance if item deleted. Corrected item–total correlations were used to assess the association between individual items and the overall scale score, while Cronbach’s alpha if item deleted was examined to evaluate the contribution of each item to overall internal consistency. Criterion validity was examined through receiver operating characteristic (ROC) analyses. Sensitivity, specificity, positive and negative predictive values (PPV and NPV), as well as positive and negative likelihood ratios (LR+ and LR–), were calculated for a range of cut-off scores.

ROC curves were generated for each instrument, and the area under the curve (AUC) was computed to provide an overall measure of the ability of each scale to correctly distinguish cases of anxiety disorders. Optimal cut-off scores were determined based on the balance between sensitivity and specificity, taking into account the intended screening purpose of the instrument. All statistical analyses were conducted using IBM SPSS Statistics for Windows, Version 31.0 (21).

## 3 Results

Of the 1,756 patients who visited their GP, 152 declined to participate. The remaining 1,604 patients were invited to complete the GAD-7 and GAD-2, of whom 1,585 returned completed questionnaires, yielding a response rate of 91.3% (range across 24 primary care practices: 86.3–93.7%). Of these, 100 individuals either declined the follow-up MINI interview or could not be reached after three telephone attempts within two weeks and were therefore excluded. Thus, 1,485 patients completed the MINI interview and were included in prevalence analyses.

As shown in **Table 1**, the MINI-assessed sample (n = 1,485) was predominantly female (69.5%), with over half of participants aged 50 years or older. Most had secondary education (57.4%), were employed (53.2%), and were married or cohabiting (61.4%).

**Table 1:** Demographic characteristics of the study sample (n =1485).

Demographic characteristics	n	%
Age		
18-34	211	14.2
35-49	462	31.1
50-64	354	23.8
65+	458	30.8
Sex		
Male	453	30.5
Female	1032	69.5
Education		
Higher and unfinished higher education	442	29.9
General or vocational secondary and unfinished secondary	848	57.4
9-year basic, unfinished basic	187	12.7
Employment status		
Employed	787	53.2
Unemployed	84	5.7
Economically inactive	607	41.1
Marital status		
Married, cohabiting	907	61.4
Single	144	9.7
Live separately, divorced, widowed	427	28.9
Place of residence		
Capital (Riga)	309	20.8
Other city	702	47.3
Rural	474	31.9

The majority of participants lived in urban areas (68.1%). According to the MINI diagnostic assessment, the most prevalent anxiety disorders were agoraphobia (8.0%) and GAD (6.1%), followed by social phobia (4.7%), whereas OCD (1.3%), PTSD (0.9%) and panic disorder (0.7%) were comparatively rare (**Table 2**). While prevalence estimates were based on the full MINI-assessed sample (n = 1,485), the questionnaires of 18 patients had to be discarded due to insufficient data quality. Therefore, the final analytical sample for reliability and diagnostic accuracy analyses comprised 1,467 participants. The study recruitment and participant inclusion process, including eligibility assessment and exclusions, are summarized in a participant flowchart presented in **Supplementary Figure 1**. Internal consistency demonstrated good reliability for the GAD-7 (Cronbach’s α = 0.86) and acceptable reliability for the GAD-2 (Cronbach’s α = 0.72). Due to the limited number of participants meeting criteria for panic disorder, OCD and PTSD, these subtypes were excluded from further validation analyses.

**Table 2:** Prevalence of anxiety disorders established by the mini international neuropsychiatric interview in the total sample, n = 1485.

Disorder	n	% (95% CI)
Any anxiety disorder	235	15.8% (14.1-17.8)
Agoraphobia	119	8.0% (6.6–9.4)
Generalized anxiety disorder	91	6.1% (4.9–7.3)
Social phobia	70	4.7% (3.6–5.8)
Post-traumatic stress disorder	13	0.9% (0.4–1.4)
Panic disorder	11	0.7% (0.3–1.1)
Obsessive-compulsive disorder	20	1.3% (0.7-1.9)

Item-level analysis demonstrated satisfactory internal consistency for both GAD-7 and GAD-2. Corrected item–total correlations for the GAD-7 ranged from 0.54 to 0.72, indicating moderate to strong associations between individual items and the overall scale score. Cronbach’s alpha if item deleted ranged from 0.83 to 0.86, suggesting that removal of any individual item did not substantially improve internal consistency. For the GAD-2, corrected item–total correlations were 0.57 for both items, indicating acceptable internal consistency of the brief screening version. Mean item scores for the GAD-7 ranged from 0.28 to 1.03, with the highest score observed for Item 1 (“Feeling nervous, anxious or on edge”) and the lowest for Item 5 (“Being so restless that it is hard to sit still”). Detailed item-level descriptive statistics and psychometric properties are presented in **Table 3**.

**Table 3:** Item-level psychometric properties and descriptive statistics of the GAD-7 and GAD-2 in the full sample (n = 1,467).

Item	Mean/SD	Variance	Corrected item–total correlation	Cronbach’s alpha if item deleted
GAD-7				
1. Feeling nervous, anxious or on edge	1.03/0.85	0.73	0.68	0.84
2. Not being able to stop or control worrying	0.43/0.73	0.53	0.72	0.83
3. Worrying too much about different things	0.76/0.86	0.74	0.67	0.84
4. Trouble relaxing	0.50/0.78	0.61	0.68	0.84
5. Being so restless that it is hard to sit still	0.28/0.60	0.36	0.58	0.85
6. Becoming easily annoyed or irritable	0.70/0.81	0.65	0.54	0.86
7. Feeling afraid as if something awful might happen	0.42/0.71	0.50	0.59	0.85
GAD-2				
1. Feeling nervous, anxious or on edge	1.03/0.85	0.73	0.57	—
2. Not being able to stop or control worrying	0.43/0.73	0.53	0.57	—

**Figure 1:**
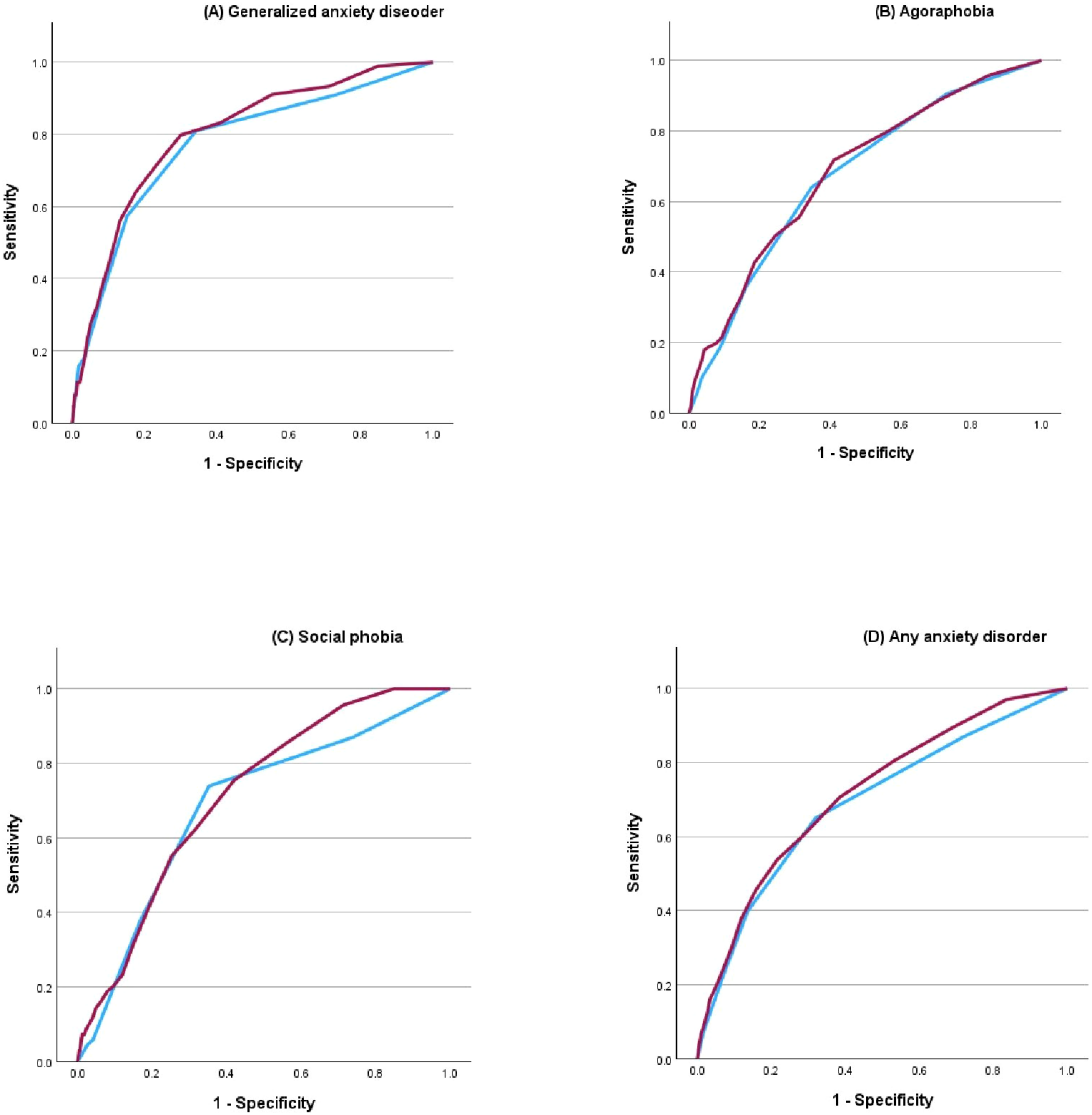
Receiver operating characteristic curves of the GAD-7 and GAD-2 for detection of MINI-defined anxiety disorders. **(A)** Generalized anxiety disorder; **(B)** Agoraphobia; **(C)** Social phobia; **(D)** Any anxiety disorder. Receiver operating characteristic curves of the GAD-7 and GAD-2. **(A)** Generalized anxiety disorder (area under the curve 0.799 and 0.772, respectively); **(B)** agoraphobia (0.685 and 0.675); **(C)** social phobia (0.715 and 0.687); **(D)** any anxiety disorder (0.718 and 0.691).

The diagnostic performance of the GAD-7 and GAD-2 across multiple cut-off thresholds for MINI-defined anxiety disorders is presented in **Table 4** and **Supplementary Table 1**. For GAD, the GAD-7 demonstrated balanced screening performance at a threshold of ≥5, with a sensitivity of 79.8% and specificity of 70.0%, whereas the GAD-2 showed comparable performance at a threshold of ≥2, yielding a sensitivity of 80.9% and specificity of 65.8%. For agoraphobia, balanced discrimination was observed at GAD-7 ≥4 (sensitivity 71.8%, specificity 59.1%) and GAD-2 ≥2 (sensitivity 64.1%, specificity 65.3%). Similarly, for social phobia, GAD-7 ≥4 demonstrated a sensitivity of 75.4% and specificity of 58.1%, while GAD-2 ≥2 yielded a sensitivity of 73.9% and specificity of 64.7%. For any MINI-defined anxiety disorder, the most clinically appropriate screening balance was observed at GAD-7 ≥4 (sensitivity 70.6%, specificity 61.6%) and GAD-2 ≥2 (sensitivity 64.9%, specificity 68.1%). **Figure 1** presents the ROC curves and corresponding AUC values for the GAD-7 and GAD-2 against MINI-defined diagnoses. The AUC was 0.799 for the GAD-7 and 0.772 for the GAD-2 for generalized anxiety disorder, 0.685 and 0.675 for agoraphobia, 0.715 and 0.687 for social phobia, and 0.718 and 0.691 for any anxiety disorder, respectively.

**Table 4:** Diagnostic performance of the GAD-7 and GAD-2 for MINI-defined anxiety disorders (n = 1,467).

Disorder & Cut-off	Sensitivity (95% CI)	Specificity (95% CI)	LR+ (95% CI)	LR– (95% CI)
Agoraphobia, n = 117				
GAD-7≥ 3	79.48 (71.29-86.07)	44.37 (41.74-47.03)	1.43 (1.29-1.58)	0.46 (0.32-0.66)
**GAD-7≥ 4**	**71.79 (63.05-79.15)**	**58.96 (56.32-61.56)**	**1.75 (1.54-1.99)**	**0.48 (0.36-0.64)**
GAD-7≥ 6	50.43 (41.50-59.33)	75.48 (73.12-77.70)	2.06 (1.98-2.14)	0.66 (0.63-0.68)
GAD-7≥ 7	42.74 (34.14-51.79)	81.48 (79.32-83.46)	2.31 (2.17-2.45)	0.70 (0.68-0.72)
GAD-7≥ 8	32.48 (24.67-41.40)	85.56 (83.58-87.33)	2.25 (2.00-2.53)	0.79 (0.77-0.81)
GAD-7≥ 9	26.50 (19.34-35.15)	88.74 (86.94-90.32)	2.35 (1.95-2.84)	0.83 (0.81-0.85)
GAD-7≥ 10	21.37 (14.91-29.64)	90.81 (89.16-92.24)	2.33 (1.72-3.15)	0.87 (0.85-0.88)
**GAD-2≥ 2**	**64.10 (55.09-72.22)**	**65.26 (62.68-67.75)**	**1.85 (1.81-1.88)**	**0.55 (0.52-0.58)**
GAD-2≥ 3	35.90 (27.78-44.91)	83.85 (81.79-85.72)	2.22 (2.03-2.44)	0.76 (0.74-0.79)
GAD-2≥ 4	18.80 (12.76-26.83)	91.11 (89.47-92.51)	2.12 (1.42-3.16)	0.89 (0.87-0.91)
Generalized anxiety disorder, n=89				
GAD-7≥ 3	91.01 (83.25-95.37)	44.63 (42.02-47.27)	1.64 (1.52-1.78)	0.20 (0.10-0.39)
GAD-7≥ 4	83.15 (74.04-89.51)	59.07 (56.45-61.64)	2.03 (1.81-2.27)	0.29 (0.18-0.45)
GAD-7≥ 5	79.78 (70.28-86.81)	69.96 (67.48-72.32)	2.66 (2.62-2.69)	0.29 (0.26-0.32)
**GAD-7≥ 6**	**71.91 (61.82-80.19)**	**76.34 (74.03-78.51)**	**3.04 (2.99-3.10)**	**0.37 (0.34-0.40)**
GAD-7≥ 7	64.04 (53.69-73.24)	82.37 (80.26-84.29)	3.63 (3.53-3.73)	0.44 (0.41-0.46)
GAD-7≥ 8	56.18 (45.83-66.02)	86.72 (84.83-88.41)	4.23 (4.06-4.41)	0.51 (0.48-0.53)
GAD-7≥ 9	44.94 (35.03-55.27)	89.62 (87.90-91.12)	4.33 (4.02-4.66)	0.61 (0.59-0.64)
GAD-7≥ 10	38.20 (28.79-48.59)	91.65 (90.08-93.00)	4.58 (4.10-5.11)	0.67 (0.65-0.70)
**GAD-2≥ 2**	**80.90 (71.52-87.72)**	**65.75 (63.20-68.21)**	**2.36 (2.34-2.39)**	**0.29 (0.26-0.33)**
GAD-2 ≥ 3	57.30 (46.93-67.07)	84.83 (82.84-86.63)	3.78 (3.64-3.93)	0.50 (0.48-0.53)
GAD-2 ≥ 4	34.83 (25.75-45.17)	91.94 (90.39-93.27)	4.32 (3.77-4.95)	0.71 (0.69-0.73)
Social phobia, n=69				
GAD-7≥ 3	85.51 (75.34-91.93)	43.85 (41.27-46.46)	1.52 (1.37-1.70)	0.33 (0.19-0.59)
GAD-7≥ 4	75.36 (64.04-84.01)	58.08 (55.48-60.64)	1.80 (1.55-2.09)	0.42 (0.28-0.64)
**GAD-7≥ 5**	**62.32 (50.52-72.82)**	**68.38 (65.90-70.77)**	**1.97 (1.91-2.04)**	**0.55 (0.51-0.59)**
GAD-7≥ 6	55.07 (43.38-66.23)	74.82 (72.48-77.03)	2.19 (2.09-2.29)	0.60 (0.56-0.64)
GAD-7≥ 7	42.03 (31.11-53.79)	80.62 (78.46-82.60)	2.17 (1.96-2.40)	0.72 (0.68-0.76)
GAD-7≥ 8	31.88 (22.09-43.58)	84.91 (82.93-86.69)	2.11 (1.73-2.58)	0.80 (0.77-0.84)
GAD-7≥ 9	23.19 (14.81-34.40)	88.05 (86.25-89.65)	1.94 (1.28-2.95)	0.87 (0.84-0.91)
GAD-7≥ 10	20.29 (12.49-31.22)	90.34 (88.68-91.78)	2.10 (1.20-3.70)	0.88 (0.85-0.91)
**GAD-2 ≥ 2**	**73.91 (62.49-82.81)**	**64.74 (62.19-67.20)**	**2.10 (2.06-2.13)**	**0.40 (0.36-0.45)**
GAD-2 ≥ 3	37.68 (27.18-49.48)	83.26 (81.21-85.13)	2.25 (1.97-2.57)	0.75 (0.71-0.78)
GAD-2 ≥ 4	18.84 (11.35-29.61)	90.77 (89.14-92.18)	2.04 (1.05-3.97)	0.89 (0.86-0.93)
Any anxiety disorder, n=231				
GAD-7≥ 3	80.52 (74.93-85.11)	46.76 (44.00-49.55)	1.51 (1.39-1.64)	0.42 (0.32-0.55)
**GAD-7≥ 4**	**70.56 (64.39-76.06)**	**61.57 (58.83-64.24)**	**1.84 (1.65-2.05)**	**0.48 (0.39-0.59)**
GAD-7≥ 5	59.74 (53.31-65.86)	71.93 (69.36-74.36)	2.13 (2.10-2.16)	0.56 (0.55-0.57)
GAD-7≥ 6	53.68 (47.24-60.00)	78.48 (76.10-80.68)	2.49 (2.44-2.55)	0.59 (0.58-0.60)
GAD-7≥ 7	45.45 (39.16-51.90)	84.22 (82.09-86.15)	2.88 (2.79-2.98)	0.65 (0.64-0.66)
GAD-7≥ 8	37.66 (31.66-44.06)	88.19 (86.27-89.87)	3.19 (3.03-3.35)	0.71 (0.70-0.72)
GAD-7≥ 9	29.87 (24.34-36.06)	90.78 (89.03-92.27)	3.24 (2.98-3.52)	0.77 (0.76-0.78)
GAD-7≥ 10	25.11 (19.95-31.08)	92.64 (91.05-93.97)	3.41 (3.02-3.85)	0.81 (0.80-0.82)
**GAD-2 ≥ 2**	**64.94 (58.58-70.80)**	**68.12 (65.47-70.66)**	**2.04 (2.01-2.06)**	**0.51 (0.50-0.53)**
GAD-2 ≥ 3	39.83 (33.73-46.26)	86.41 (84.38-88.21)	2.93 (2.80-3.06)	0.70 (0.69-0.71)
GAD-2 ≥ 4	22.94 (17.99-28.78)	92.80 (91.22-94.11)	3.19 (2.75-3.69)	0.83 (0.82-0.84)

Agoraphobia, generalized anxiety disorder, social phobia, and any anxiety disorder. Bold values indicate the selected optimal cut-off point based on the balance between sensitivity and specificity.

## 4 Discussion

This study represents the large-scale validation of the GAD-7 and GAD-2 for different subtypes of anxiety disorders in a Latvian primary care population using the MINI as a structured diagnostic reference standard. Both instruments demonstrated good internal consistency and clinically meaningful diagnostic accuracy for identifying GAD and a weaker propensity to detect other subtypes of anxiety disorders within this setting.

The prevalence of any anxiety disorder in our study was 15.8%, encompassing panic disorder, agoraphobia, social phobia, post-traumatic stress disorder, and GAD. This estimate is lower than that reported in Madrid (22.4%) (22) and slightly below Spanish 12-month primary care rates (18.49%) (23), yet comparable to findings from Israel (19.4%) (24) and Belgium (16.1%) (25). Taken together, these comparisons suggest that the burden of anxiety disorders in Latvian primary care is broadly consistent with other European settings, with a diagnostic spectrum comparable to that reported in similar primary care populations (23, 26, 27).

The internal consistency coefficients indicate good reliability for the GAD-7 and acceptable reliability for the GAD-2 in this population. The Cronbach’s alpha for the GAD-7 is consistent with values reported in international validation studies, supporting the structural integrity of the scale in Latvian primary care (12, 13, 15). As expected for a two-item instrument, the GAD-2 yielded a lower alpha coefficient; nevertheless, its reliability remains adequate for a brief screening tool intended for routine clinical use. The item-level analyses additionally supported the internal homogeneity of the Latvian version of the GAD-7, with all items demonstrating satisfactory corrected item–total correlations and stable internal consistency following individual item deletion analyses.

With respect to diagnostic discrimination, AUC estimates demonstrated the strongest performance for GAD (GAD-7 AUC = 0.799; GAD-2 AUC = 0.772), acceptable for social phobia (0.715 and 0.687), and comparatively lower discrimination for agoraphobia (0.685 and 0.675) (28). For any anxiety disorder, AUC values were 0.718 for the GAD-7 and 0.691 for the GAD-2, indicating acceptable overall discrimination across the broader anxiety spectrum. Consistent with these findings, sensitivity and specificity demonstrated a more favorable balance for GAD compared with agoraphobia and social phobia, suggesting stronger screening performance for generalized anxiety symptoms than for non-generalized anxiety phenotypes. Conceptually, this gradient of diagnostic accuracy is consistent with the original construct of the GAD-7, which primarily assesses generalized worry, excessive anxiety, and somatic tension rather than disorder-specific symptoms such as avoidance behavior, panic-related symptoms, or performance-related fears (6, 29). Consequently, anxiety disorders characterized predominantly by anticipatory avoidance, situational fear, or social-evaluative concerns may be less effectively captured by the GAD-7 and GAD-2. This symptom-domain mismatch may partly explain the comparatively lower sensitivity and AUC values observed for agoraphobia and social phobia in the present study.

The optimal thresholds for GAD (GAD-7 ≥5; GAD-2 ≥2) were lower than those proposed in the original validation studies (6, 9). In primary care populations, lower cut-off values may increase sensitivity and improve early identification of clinically relevant anxiety symptoms, although this typically occurs at the expense of specificity (11, 30, 31). Therefore, optimal thresholds should be interpreted within the context of the intended screening purpose and characteristics of the target population rather than as universally fixed parameters.

Compared with the meta-analysis by Aktürk et al. (2025), which evaluated the GAD-7 and GAD-2 mainly at thresholds of ≥10 and ≥3, respectively, our analyses indicated lower optimal cut-offs (≥5 and ≥2) for detecting both GAD and any anxiety disorder in this primary care population (30).

Similarly, Park and Park (2025) reported pooled sensitivity and specificity of 0.81 and 0.78 for the GAD-7 (sROC AUC 0.87), and 0.78 and 0.81 for the GAD-2 (sROC AUC 0.86) across diverse international samples (31).

Although AUC values in our Latvian cohort were somewhat lower, they followed a comparable hierarchical pattern, with the highest discrimination for GAD and attenuated performance for other anxiety subtypes. The modest reduction in AUC values relative to pooled international estimates, as well as the lower optimal cut-off values identified in the present study, likely reflect several interacting factors, including country- and language-specific influences, differences in case mix, and the clinical heterogeneity inherent to routine primary care populations (11, 15, 30, 31). Anxiety symptoms in primary care settings are often less severe, more heterogeneous, and more likely to overlap with somatic complaints than in psychiatric samples (2, 4, 6, 32). In addition, the lower prevalence of some MINI-defined anxiety disorders may have influenced threshold optimization and reduced the stability of disorder-specific estimates. Lower screening thresholds generally prioritize sensitivity over specificity, which may be clinically preferable in primary care settings focused on early case identification and subsequent diagnostic assessment (11, 30, 31). Finally, cultural and language-related differences in symptom perception, reporting style, and interpretation of questionnaire items may also contribute to variability in optimal screening thresholds across countries and populations. Previous cross-cultural psychometric research has demonstrated that even relatively small translation differences may introduce localized measurement variation and influence item functioning, underscoring the importance of careful linguistic adaptation and population-specific validation (16, 19).

Another possible explanation for the lower AUC values observed in the present study may relate to variability in the reference standard assessment. Although all MINI interviews were conducted by trained psychiatrists who were blinded to GAD-7 scores, formal inter-rater reliability across the four interviewers was not assessed. Therefore, potential differences in diagnostic interviewing style or threshold application cannot be fully excluded. However, previous validation studies have demonstrated good reliability of the MINI when administered by trained interviewers. The original MINI validation studies reported satisfactory inter-rater and test–retest reliability in comparison with the Composite International Diagnostic Interview and the Structured Clinical Interview for DSM Disorders (17, 33). Subsequent language-specific validation studies have confirmed these findings; for example, the Italian version demonstrated high inter-rater reliability, with kappa coefficients exceeding 0.73 across diagnostic categories (34), while the Japanese version showed excellent agreement for several anxiety and mood disorders, with kappa coefficients generally exceeding 0.75 (35). Nevertheless, the absence of a study-specific assessment of inter-rater reliability remains a limitation of the present study. In addition, the GAD-7 assesses symptoms during the preceding two weeks, whereas anxiety symptoms may fluctuate over time, potentially affecting agreement between self-reported symptom severity and MINI-based diagnostic classification.

In a Finnish primary care validation study of the GAD-7, a cut-off score of ≥7 yielded a sensitivity of 100% and a specificity of 82.6% for detecting generalized anxiety disorder, while a lower threshold of ≥5 achieved a sensitivity of 80% and a specificity of 73.6% for identifying other anxiety disorders (15).

Consistent with this threshold-dependent pattern, our findings demonstrate that lower cut-offs enhance sensitivity in primary care settings, supporting their potential utility in early case identification when followed by structured diagnostic assessment (6, 29). From a clinical perspective, these findings further support the use of the GAD-7 and GAD-2 primarily as broad transdiagnostic screening instruments for generalized anxiety symptoms in primary care rather than as disorder-specific detection tools for all anxiety disorder subtypes. Additional assessment instruments or targeted clinical evaluation may therefore be necessary to improve detection of avoidance-based anxiety disorders within routine primary care settings.

## 5 Strengths and limitations

This study has several notable strengths. The sample consisted exclusively of primary care patients recruited from practices across all regions of Latvia, including both urban and rural settings, thereby enhancing generalizability. Participants who consented to the diagnostic assessment underwent evaluation using the MINI as a structured reference standard, and interviews were conducted by trained psychiatrists who were blinded to GAD-7 scores, thereby minimizing assessment bias.

However, several limitations should be acknowledged. Due to the small number of cases, disorder-specific cut-off values were not calculated for panic disorder, posttraumatic stress disorder, or OCD. Consequently, the diagnostic performance of the GAD-7 and GAD-2 for these conditions should be interpreted with caution. This limits conclusions regarding the performance of the GAD-7 and GAD-2 across the full spectrum of anxiety disorders. Furthermore, the comparatively lower diagnostic performance observed for certain anxiety disorder subtypes indicates that the GAD-7 and GAD-2 may function more effectively as broad anxiety screening instruments rather than disorder-specific diagnostic tools in primary care populations. The cross-sectional design precludes conclusions regarding temporal stability or causal relationships. In addition, because the MINI interviews were conducted within two weeks of the initial GAD-7 assessment, fluctuations in anxiety symptom severity over time may have influenced agreement between self-reported symptoms and MINI-based diagnostic classification. Formal inter-rater reliability of MINI assessments across the four psychiatrists was not evaluated, which may have contributed to variability in diagnostic classification and partly influenced the lower AUC values observed in comparison with some previous studies. Potential nonresponse bias should also be considered, as some eligible participants could not be reached or did not complete the MINI interview. The predominance of female participants may limit the generalizability of the findings to broader primary care populations. Finally, because the GAD-7 and GAD-2 are self-report instruments, responses may have been influenced by recall bias or individual differences in symptom perception and reporting. Further research in other clinical populations is warranted to refine sensitivity, specificity, and optimal cut-off thresholds, particularly in samples with different case mix and prevalence profiles.

## 6 Conclusion

This study provides the large-scale evaluation of the GAD-7 and GAD-2 for different subtypes of anxiety disorders in Latvian primary care using a structured diagnostic reference standard. Both instruments demonstrated good reliability and clinically meaningful diagnostic accuracy, with strongest performance observed for generalized anxiety disorder, whereas discrimination for other anxiety disorder subtypes was comparatively more limited.

These findings indicate that the GAD-7 and GAD-2 are appropriate tools for screening generalized anxiety disorder in routine primary care, while their performance for other anxiety disorders should be interpreted with consideration of subtype-specific characteristics. The results underscore the importance of context-sensitive cut-off calibration and support the use of these instruments as part of a stepped diagnostic approach in primary care.

## Data Availability

The raw data supporting the conclusions of this article will be made available by the authors, without undue reservation.

## Supplementary material

Supplementary Figure 1 Participant flowchart of study recruitment and inclusion process. **Figure 1** presents the flow of participants through the study, including recruitment, eligibility assessment, exclusion criteria, and final inclusion in the statistical analyses.
