# Supplementary figures and images for: The reliability and diagnostic accuracy of the GAD-7 and GAD-2 for the most prevalent anxiety disorders in Latvian primary care
Source: Front Psychiatry. 2026 Jul 7;17:1855491. doi: 10.3389/fpsyt.2026.1855491 (PMC13385674; doi:10.3389/fpsyt.2026.1855491)

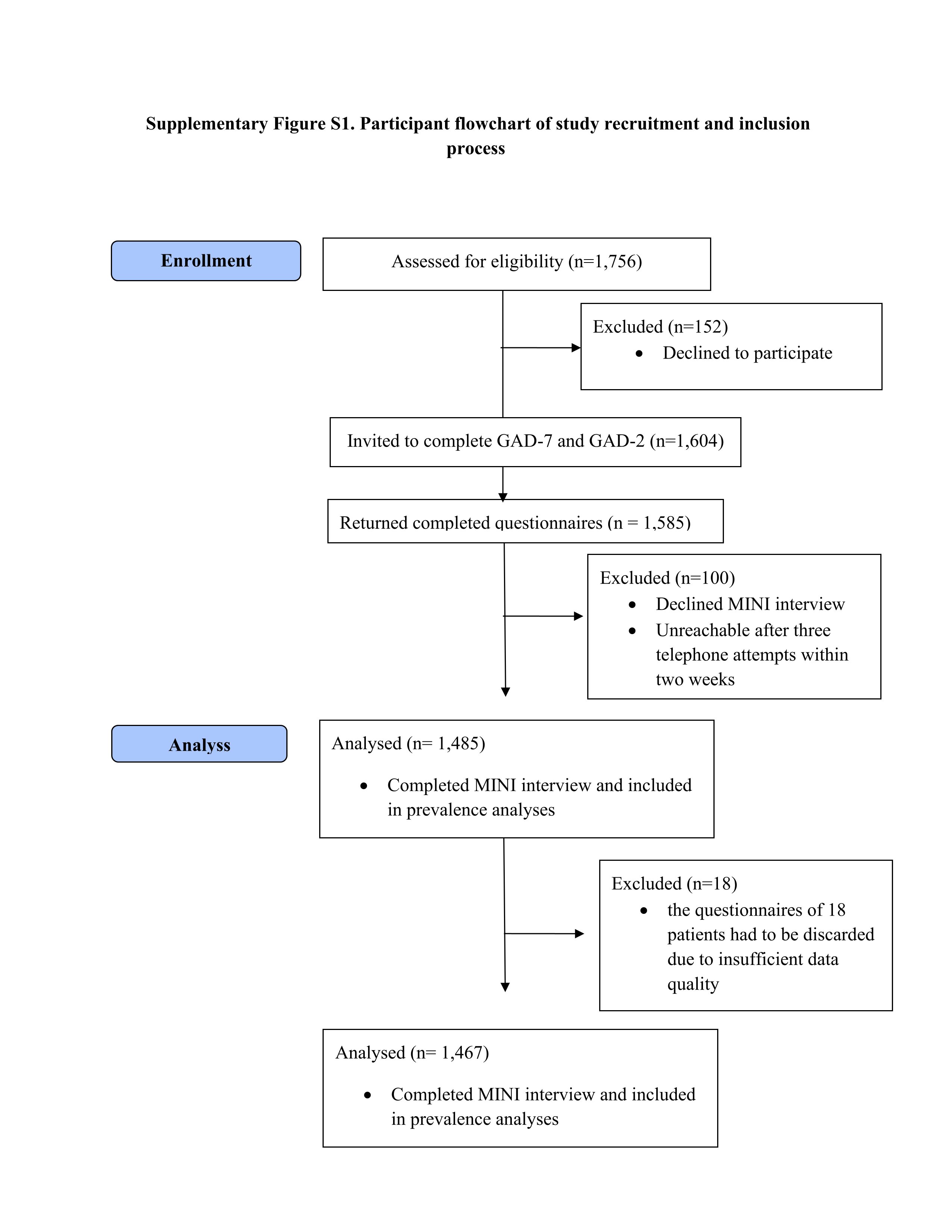

Supplement: Supplementary Figure 1 — Participant flowchart of study recruitment and inclusion process. Figure 1 presents the flow of participants through the study, including recruitment, eligibility assessment, exclusion criteria, and final inclusion in the statistical analyses. [file Image1.jpeg]
