# Supplementary material for: The reliability and diagnostic accuracy of the GAD-7 and GAD-2 for the most prevalent anxiety disorders in Latvian primary care
Source: Front Psychiatry. 2026 Jul 7;17:1855491. doi: 10.3389/fpsyt.2026.1855491 (PMC13385674; doi:10.3389/fpsyt.2026.1855491)
Supplement: Supplementary file 2 [file Table1.docx]

| **Supplementary Table S1. Full diagnostic performance of the GAD-7 and GAD-2 across cut-off thresholds for MINI-defined anxiety disorders (n = 1,467) *Agoraphobia, generalized anxiety disorder, social phobia, PTSD, panic disorder, and any anxiety disorder*** | | | | |
| --- | --- | --- | --- | --- |
| **Disorder &**  **Cut-off** | **Sensitivity**  **(95% CI)** | **Specificity**  **(95% CI)** | **LR+**  **(95% CI)** | **LR–**  **(95% CI)** |
| **AGORAPHOBIA, n = 117** | | | | |
| GAD-7≥ 3 | 79.48 (71.29-86.07) | 44.37 (41.74-47.03) | 1.43 (1.29-1.58) | 0.46 (0.32-0.66) |
| **GAD-7≥ 4** | **71.79 (63.05-79.15)** | **58.96 (56.32-61.56)** | **1.75 (1.54-1.99)** | **0.48 (0.36-0.64)** |
| GAD-7≥ 5 | 55.56 (46.52-64.24) | 68.89 (66.37-71.30) | 1.79 (1.74-1.84) | 0.65 (0.62-0.67) |
| GAD-7≥ 6 | 50.43 (41.50-59.33) | 75.48 (73.12-77.70) | 2.06 (1.98-2.14) | 0.66 (0.63-0.68) |
| GAD-7≥ 7 | 42.74 (34.14-51.79) | 81.48 (79.32-83.46) | 2.31 (2.17-2.45) | 0.70 (0.68-0.72) |
| GAD-7≥ 8 | 32.48 (24.67-41.40) | 85.56 (83.58-87.33) | 2.25 (2.00-2.53) | 0.79 (0.77-0.81) |
| GAD-7≥ 9 | 26.50 (19.34-35.15) | 88.74 (86.94-90.32) | 2.35 (1.95-2.84) | 0.83 (0.81-0.85) |
| GAD-7≥ 10 | 21.37 (14.91-29.64) | 90.81 (89.16-92.24) | 2.33 (1.72-3.15) | 0.87 (0.85-0.88) |
| GAD-7≥ 11 | 19.66 (13.47-27.77) | 92.52 (90.99-93.80) | 2.63 (1.74-3.97) | 0.87 (0.79-0.95) |
| GAD-7≥ 12 | 18.80 (12.76-26.83) | 94.44 (93.09-95.54) | 3.38 (2.19-5.23) | 0.86 (0.79-0.94) |
| GAD-7≥ 13 | 17.95 (12.05-25.88) | 95.70 (94.49-96.66) | 4.18 (2.63-6.63) | 0.86 (0.79-0.93) |
| GAD-7≥ 14 | 14.53 (9.27-22.04) | 96.59 (95.49-97.44) | 4.26 (2.53-7.20) | 0.88 (0.82-0.95) |
| GAD-7≥ 15 | 10.25 (5.97-17.07) | 98.07 (97.19-98.68) | 5.33 (2.76-10.28) | 0.92 (0.86-0.97) |
| GAD-2 ≥ 1 | 90.60 (83.95-94.67) | 26.96 (24.66-29.39) | 1.24 (1.16-1.33) | 0.35 (0.20-0.62) |
| **GAD-2 ≥ 2** | **64.10 (55.09-72.22)** | **65.26 (62.68-67.75)** | **1.85 (1.81-1.88)** | **0.55 (0.52-0.58)** |
| GAD-2 ≥ 3 | 35.90 (27.78-44.91) | 83.85 (81.79-85.72) | 2.22 (2.03-2.44) | 0.76 (0.74-0.79) |
| GAD-2 ≥ 4 | 18.80 (12.76-26.83) | 91.11 (89.47-92.51) | 2.12 (1.42-3.16) | 0.89 (0.87-0.91) |
| GAD-2 ≥ 5 | 10.26 (5.97-17.07) | 96.37 (95.23-97.24) | 2.83 (1.55-5.16) | 0.93 (0.88-0.99) |
| GAD-2 ≥ 6 | 5.98 (2.93-11.84) | 97.70 (96.76-98.38) | 2.61 (1.17-5.79) | 0.96 (0.92-1.01) |
| **GENERALIZED ANXIETY DISORDER, n=89** | | | | |
| GAD-7≥ 3 | 91.01 (83.25-95.37) | 44.63 (42.02-47.27) | 1.64 (1.52-1.78) | 0.20 (0.10-0.39) |
| GAD-7≥ 4 | 83.15 (74.04-89.51) | 59.07 (56.45-61.64) | 2.03 (1.81-2.27) | 0.29 (0.18-0.45) |
| GAD-7≥ 5 | 79.78 (70.28-86.81) | 69.96 (67.48-72.32) | 2.66 (2.62-2.69) | 0.29 (0.26-0.32) |
| **GAD-7≥ 6** | **71.91 (61.82-80.19)** | **76.34 (74.03-78.51)** | **3.04 (2.99-3.10)** | **0.37 (0.34-0.40)** |
| GAD-7≥ 7 | 64.04 (53.69-73.24) | 82.37 (80.26-84.29) | 3.63 (3.53-3.73) | 0.44 (0.41-0.46) |
| GAD-7≥ 8 | 56.18 (45.83-66.02) | 86.72 (84.83-88.41) | 4.23 (4.06-4.41) | 0.51 (0.48-0.53) |
| GAD-7≥ 9 | 44.94 (35.03-55.27) | 89.62 (87.90-91.12) | 4.33 (4.02-4.66) | 0.61 (0.59-0.64) |
| GAD-7≥ 10 | 38.20 (28.79-48.59) | 91.65 (90.08-93.00) | 4.58 (4.10-5.11) | 0.67 (0.65-0.70) |
| GAD-7≥ 11 | 32.58 (23.74-42.87) | 93.11 (91.65-94.33) | 6.24 (4.29-9.06) | 0.71 (0.62-0.82) |
| GAD-7≥ 12 | 28.09 (19.81-38.18) | 94.78 (93.47-95.83) | 6.67 (4.40-10.13) | 0.75 (0.66-0.86) |
| GAD-7≥ 13 | 23.60 (15.98-33.39) | 95.79 (94.60-96.73) | 5.61 (3.57-8.80) | 0.80 (0.71-0.90) |
| GAD-7≥ 14 | 17.98 (11.38-27.22) | 96.59 (95.49-97.43) | 5.27 (3.12-8.91) | 0.85 (0.77-0.94) |
| GAD-7≥ 15 | 11.24 (6.22-19.46) | 97.97 (97.08-98.59) | 5.53 (2.77-11.02) | 0.91 (0.84-0.98) |
| GAD-2 ≥ 1 | 91.01 (83.25-95.37) | 26.63 (24.37-29.03) | 1.24 (1.15-1.33) | 0.34 (0.17-0.66) |
| **GAD-2 ≥ 2** | **80.90 (71.52-87.72)** | **65.75 (63.20-68.21)** | **2.36 (2.34-2.39)** | **0.29 (0.26-0.33)** |
| GAD-2 ≥ 3 | 57.30 (46.93-67.07) | 84.83 (82.84-86.63) | 3.78 (3.64-3.93) | 0.50 (0.48-0.53) |
| GAD-2 ≥ 4 | 34.83 (25.75-45.17) | 91.94 (90.39-93.27) | 4.32 (3.77-4.95) | 0.71 (0.69-0.73) |
| GAD-2 ≥ 5 | 17.98 (11.38-27.22) | 96.73 (95.66-97.55) | 5.51 (3.24-9.34) | 0.85 (0.77-0.93) |
| GAD-2 ≥ 6 | 15.73 (9.61-24.69) | 98.26 (97.42-98.83) | 9.03 (4.84-16.85) | 0.86 (0.78-0.94) |
| **SOCIAL PHOBIA n=69** | | | | |
| GAD-7≥ 3 | 85.51 (75.34-91.93) | 43.85 (41.27-46.46) | 1.52 (1.37-1.70) | 0.33 (0.19-0.59) |
| GAD-7≥ 4 | 75.36 (64.04-84.01) | 58.08 (55.48-60.64) | 1.80 (1.55-2.09) | 0.42 (0.28-0.64) |
| **GAD-7≥ 5** | **62.32 (50.52-72.82)** | **68.38 (65.90-70.77)** | **1.97 (1.91-2.04)** | **0.55 (0.51-0.59)** |
| GAD-7≥ 6 | 55.07 (43.38-66.23) | 74.82 (72.48-77.03) | 2.19 (2.09-2.29) | 0.60 (0.56-0.64) |
| GAD-7≥ 7 | 42.03 (31.11-53.79) | 80.62 (78.46-82.60) | 2.17 (1.96-2.40) | 0.72 (0.68-0.76) |
| GAD-7≥ 8 | 31.88 (22.09-43.58) | 84.91 (82.93-86.69) | 2.11 (1.73-2.58) | 0.80 (0.77-0.84) |
| GAD-7≥ 9 | 23.19 (14.81-34.40) | 88.05 (86.25-89.65) | 1.94 (1.28-2.95) | 0.87 (0.84-0.91) |
| GAD-7≥ 10 | 20.29 (12.49-31.22) | 90.34 (88.68-91.78) | 2.10 (1.20-3.70) | 0.88 (0.85-0.91) |
| GAD-7≥ 11 | 18.84 (11.36-29.61) | 92.06 (90.52-93.36) | 2.37 (1.41-4.00) | 0.88 (0.79-0.99) |
| GAD-7≥ 12 | 15.94 (9.14-26.33) | 93.85 (92.46-94.99) | 2.59 (1.45-4.62) | 0.90 (0.81-0.99) |
| GAD-7≥ 13 | 14.49 (8.07-24.66) | 95.06 (93.80-96.08) | 2.94 (1.58-5.45) | 0.90 (0.82-0.99) |
| GAD-7≥ 14 | 11.59 (5.99-21.25) | 96.07 (94.91-96.97) | 2.95 (1.46-5.94) | 0.92 (0.84-1.00) |
| GAD-7≥ 15 | 8.70 (4.05-17.70) | 97.71 (96.79-98.37) | 3.80 (1.64-8.78) | 0.93 (0.87-1.01) |
| GAD-2 ≥ 1 | 86.96 (77.03-92.98) | 26.18 (23.94-28.55) | 1.18 (1.07-1.30) | 0.50 (0.27-0.92) |
| **GAD-2 ≥ 2** | **73.91 (62.49-82.81)** | **64.74 (62.19-67.20)** | **2.10 (2.06-2.13)** | **0.40 (0.36-0.45)** |
| GAD-2 ≥ 3 | 37.68 (27.18-49.48) | 83.26 (81.21-85.13) | 2.25 (1.97-2.57) | 0.75 (0.71-0.78) |
| GAD-2 ≥ 4 | 18.84 (11.35-29.61) | 90.77 (89.14-92.18) | 2.04 (1.05-3.97) | 0.89 (0.86-0.93) |
| GAD-2 ≥ 5 | 5.80 (2.28-13.98) | 96.54 (95.45-97.38) | 1.42 (0.53-3.81) | 0.98 (0.93-1.04) |
| GAD-2 ≥ 6 | 4.35 (1.49-12.02) | 97.50 (96.54-98.19) | 1.74 (0.55-5.51) | 0.98 (0.93-1.03) |
| **ANY ANXIETY n=231** | | | | |
| GAD-7≥ 3 | 80.52 (74.93-85.11) | 46.76 (44.00-49.55) | 1.51 (1.39-1.64) | 0.42 (0.32-0.55) |
| **GAD-7≥ 4** | **70.56 (64.39-76.06)** | **61.57 (58.83-64.24)** | **1.84 (1.65-2.05)** | **0.48 (0.39-0.59)** |
| GAD-7≥ 5 | 59.74 (53.31-65.86) | 71.93 (69.36-74.36) | 2.13 (2.10-2.16) | 0.56 (0.55-0.57) |
| GAD-7≥ 6 | 53.68 (47.24-60.00) | 78.48 (76.10-80.68) | 2.49 (2.44-2.55) | 0.59 (0.58-0.60) |
| GAD-7≥ 7 | 45.45 (39.16-51.90) | 84.22 (82.09-86.15) | 2.88 (2.79-2.98) | 0.65 (0.64-0.66) |
| GAD-7≥ 8 | 37.66 (31.66-44.06) | 88.19 (86.27-89.87) | 3.19 (3.03-3.35) | 0.71 (0.70-0.72) |
| GAD-7≥ 9 | 29.87 (24.34-36.06) | 90.78 (89.03-92.27) | 3.24 (2.98-3.52) | 0.77 (0.76-0.78) |
| GAD-7≥ 10 | 25.11 (19.95-31.08) | 92.64 (91.05-93.97) | 3.41 (3.02-3.85) | 0.81 (0.80-0.82) |
| GAD-7≥ 11 | 21.65 (16.82-27.40) | 94.01 (92.55-95.20) | 3.62 (2.60-5.03) | 0.83 (0.78-0.89) |
| GAD-7≥ 12 | 18.18 (13.74-23.66) | 95.55 (94.25-96.57) | 4.09 (2.80-5.95) | 0.86 (0.80-0.91) |
| GAD-7≥ 13 | 16.02 (11.85-21.30) | 96.60 (95.44-97.48) | 4.71 (3.10-7.17) | 0.87 (0.82-0.92) |
| GAD-7≥ 14 | 12.55 (8.89-17.45) | 97.25 (96.18-98.02) | 4.56 (2.84-7.34) | 0.90 (0.86-0.95) |
| GAD-7≥ 15 | 8.23 (5.33-12.49) | 98.46 (97.61-99.01) | 5.35 (2.88-9.95) | 0.93 (0.90-0.97) |
| GAD-2 ≥ 1 | 87.01 (82.07-90.75) | 27.91 (25.48-30.48) | 1.21 (1.14-1.28) | 0.47 (0.33-0.66) |
| **GAD-2 ≥ 2** | **64.94 (58.58-70.80)** | **68.12 (65.47-70.66)** | **2.04 (2.01-2.06)** | **0.51 (0.50-0.53)** |
| GAD-2 ≥ 3 | 39.83 (33.73-46.26) | 86.41 (84.38-88.21) | 2.93 (2.80-3.06) | 0.70 (0.69-0.71) |
| GAD-2 ≥ 4 | 22.94 (17.99-28.78) | 92.80 (91.22-94.11) | 3.19 (2.75-3.69) | 0.83 (0.82-0.84) |
| GAD-2 ≥ 5 | 10.82 (7.44-15.49) | 97.09 (95.99-97.89) | 3.72 (2.28-6.07) | 0.92 (0.88-0.96) |
| GAD-2 ≥ 6 | 7.36 (4.65-11.47) | 98.30 (97.42-98.89) | 4.33 (2.32-8.08) | 0.94 (0.91-0.98) |
